# “The Tragedy of the Commons”: How Individualism and Collectivism Affected the Spread of the COVID-19 Pandemic

**DOI:** 10.3389/fpubh.2021.627559

**Published:** 2021-02-11

**Authors:** Yossi Maaravi, Aharon Levy, Tamar Gur, Dan Confino, Sandra Segal

**Affiliations:** ^1^The Adelson School of Entrepreneurship, Interdisciplinary Center, Herzliya, Israel; ^2^Department of Psychology, Graduate School of Arts and Sciences, Yale University, New Haven, CT, United States; ^3^Department of Psychology, Hebrew University of Jerusalem, Jerusalem, Israel; ^4^Département de Psychologie, Université de Genève, Geneva, Switzerland

**Keywords:** COVID-19, individualism–collectivism, Hofstede, the tragedy of the commons, public adherence

## Abstract

Why did COVID-19 hit some countries harder than others? While this question is usually answered based on demographics (e. g., population age), health policy (e.g., quarantine), or economic factors, we argue that cultural variance across countries is just as crucial in understanding how susceptible a society is to the COVID-19 outbreak. To test this hypothesis, we first analyzed data collected across 69 countries and examined the relationship between culture and the impact of COVID. Next, we conducted two studies to validate our findings further and explore the mechanism at hand. As expected, we found that the more individualistic (vs. collectivistic) a country was, the more COVID-19 cases and mortalities it had. We also found that the more individualistic participants were, the higher the chances they would not adhere to epidemic prevention measures. These findings are important in understanding the spread of the pandemic, devising optimal exit strategies from lockdowns, and persuading the population to get the new vaccine against the virus.

## Introduction

In just a few months since the first cases of COVID-19 were reported in China, SARS-CoV-2 has spread to almost all countries, infecting tens of millions, killing over a million and a half people, and undermining national and global economies (1). As the World Health Organization declared COVID-19 a pandemic and announced a global emergency (2), governments across the globe have issued numerous guidelines and measures to fight the spread and avoid catastrophic consequences. Some of the most common measures include reducing human contact through quarantine, isolation, and social distancing, as well as preventing infection through wearing masks, washing hands, and sterilizing surfaces (3).

To help policy-makers mitigate the pandemic, scientists and health organizations have been investigating different factors for contagion and prevention. One interesting question that has not been fully answered is the virus differential impact across various countries. Among the most commonly discussed factors for this variance are demographic and historical factors such as age, comorbidities of the population in different countries (4), and “countries” prior experience in dealing with such pandemics in recent years–e.g., Taiwan and the SARS epidemic in 2003 (5).

In the current article, we argue that cultural dimensions may also play a role in explaining the differential effect of the pandemic across countries and should therefore be taken into account when choosing the optimal measures needed to combat COVID-19 or similar pandemics in the future. Culture is defined as “the collective programming of the mind which distinguishes the members of one category of people from another” (6). Thus, population behavior and the psychological factors behind it may depend in part on a given country's culture (7). This may be crucial in understanding both COVID-19's spread and its mitigation–e.g., adhering to health authorities' guidelines such as social distancing or wearing masks (8). Indeed, a recent review (9) has identified several social and behavioral science insights—including cultural norms–that may support COVID-19 pandemic response, and called researchers to fill possible gaps urgently.

Here, we posit that the cultural aspect of Individualism vs. Collectivism is crucial in understanding the pandemic's global pattern (10). The individualism-collectivism continuum (11) describes the degree to which individuals in a given culture see themselves as independent—vs. interdependent—of the society they live in. It translates to individuals' self-concept of “I” or “we,” which in turn, dictates how much they care for themselves and their immediate families only, as opposed to the entire community they live in, or—the larger whole.

Hardin's classic article “The Tragedy of the Commons” (12) offers a prediction for the difference between Individualistic vs. Collectivistic societies facing the pandemic. Hardin described a social dilemma where each decision-maker in a community is better off acting egocentrically. Still, if others acted likewise without concern for the cumulative impact on society, “the commons” are eventually destroyed. Indeed, subsequent literature (13) has indicated that people from different national cultures followed different decision-making schemas in such dilemmas that were dictated in part by their countries' individualistic vs. collectivistic approaches. It is relevant here, as fighting COVID-19 requires focusing on the common good [e.g., flattening the curve, (14)] more than on individualistic interests (e.g., going to work).

Interestingly, while common sense suggests that the spread of the virus will be more intensive in collectivistic societies due to their closer and more frequent social interactions, the combination of culture and Hardin's theory predict the opposite: the pandemic's impact will be greater in individualistic societies where people care less for the greater good. Thus, we hypothesized “The tragedy of individualistic societies” in facing COVID-19. Specifically, we argue and provide evidence across three studies that the spread of the pandemic and its consequences–in terms of cases and deaths—may be explained in part by the degree of societies' individualistic vs. collectivistic orientation in that the more individualistic a society is, the more it will be impacted by the pandemic.

## Study 1

In Study 1, we investigated the relationship between the individualism-collectivism dimension using Hofstede's cultural dimension model and the number of COVID-19 cases and related deaths. This was done for all 69 countries, for which data was available in Hofstede's national culture survey (version 2015 12 08). The total population in these countries is 5.87 billion, representing 75% of the entire world population.

### Methods

Information was retrieved from all databases used in Study 1 on April 21st, 2020. Hofstede's individualism score of national culture was retrieved from Hofstede's national culture survey (15). All COVID-19 related variables, i.e., number of Coronavirus cases, total tests per one million residents, and Coronavirus related deaths, were retrieved from the “Worldmeters” website, which presents constantly updating information about the SARS-CoV-2 (16). The number of days since the outbreak of Coronavirus disease in each country was calculated as the number of days since 100 people were diagnosed with the disease in the country (17). The information retrieved from this website was updated as of April 21st, 2020. The “Worldmeters” website is considered reliable and used by international agencies and academic research. Since much of the information regarding state demographic information in recent years was unavailable, with respect to each index, we used the most recent assessment that was available for the majority of the selected states in the sample (the year of the most recent assessment, i.e., the retrieved assessment, is in parenthesis). State population demographic information–i.e., percentage of population above 65, percentage of Urban Population (2018), Democracy index, Life expectancy at birth in years (2018), Population density (2018), GINI index (2016), percentage of the budget for healthcare (2017)–were all obtained from the World Bank website (18).

### Results and Discussion

**Figure 1:**
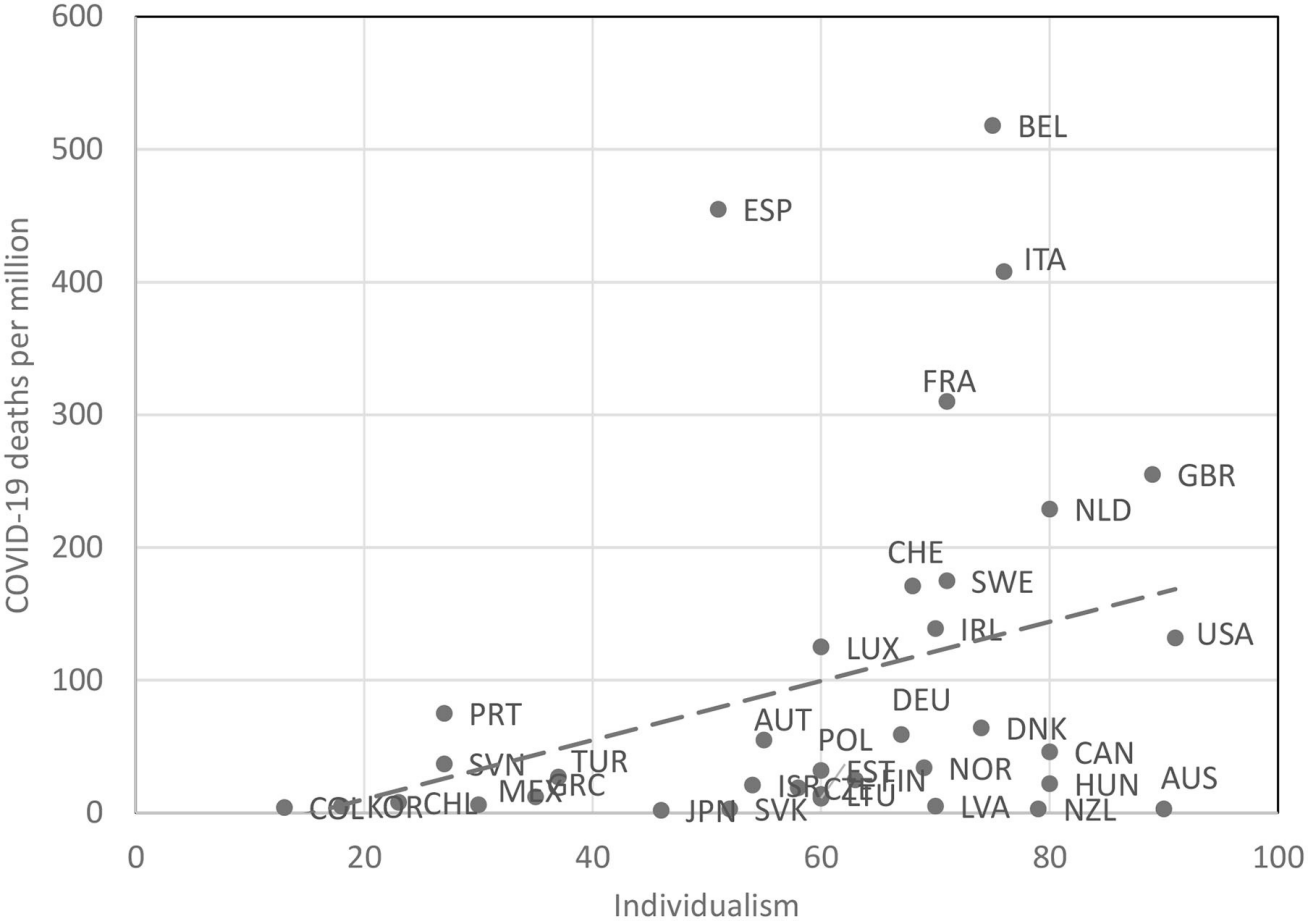
The relationship between countries' individualism score and COVID-19 deaths within a given country for OECD countries (for country codes see Supplementary Materials; Study 1).

We first conducted two simple correlations analyses to examine the association between Hofstede's Individualism score with the number of COVID-19 cases and COVID-19 related deaths. The correlations between Hofstede's Individualism score and the number of COVID-19 cases (*r* = 0.49, *p* < 0.001), and COVID-19 related deaths were highly significant (*r* = 0.48, *p* < 0.001). To compare countries with similar economic or ideological backgrounds, we then examined the association between those same variables only among the 36 OECD countries (used in our original sample). We found a similar yet nearing significant pattern of correlations between Hofstede's Individualism score and the number of COVID-19 cases among the sample of OECD countries (see Supplementary Materials; *r* = 0.29, *p* = 0.09). We also found the same pattern of correlations between Hofstede's Individualism score and the number of COVID-19 deaths among the sample of OECD countries (see Figure 1; *r* = 0.35, *p* = 0.040).

We then conducted the same correlations analyses on the complete sample while controlling for eight relevant variables, i.e., days since outbreak of the pandemic, percentage of population over 65, democracy index, Gini index, percentage of the budget for health care, life expectancy, population density and total COVID-19 tests per million. Both the correlation between Hofstede's Individualism score with the number of COVID-19 cases (*r* = 0.34, *p* = 0.028), and the correlation between Hofstede's Individualism score with the number of COVID-19 related deaths were significant when we controlled for the variables mentioned above (*r* = 0.33, *p* = 0.036). Taken together, these results suggest that, indeed, the more individualistic a society is, the more it suffers from COVID-19 related cases and deaths.

## Study 2

In Study 2, we investigated the possible mechanism for the above pattern. We picked Israel since the country scored 54 on Hofstede's individualism-collectivism model, which is approximately the mid-range of the 69 countries (6-91).

### Methods

#### Sample

Our sample consisted of 327 Israelis [49.8% women: Mage (mean age) = 44.44, SD = 14.28]. Participants were contacted via a large Israeli online survey company (iPanel) and asked to participate in exchange for monetary compensation. Most participants (98.2%) defined themselves as Jews. The rest of the participants defined themselves as either Muslim (0.6%), Christians (0.3%), Druze (0.3%), or religionless (0.6%; for additional information, see Supplementary Materials).

#### Procedure

We investigated a serial mediation model with four levels: people's norms of individualism vs. collectivism, their collectivistic attitudes, their COVID-19 planned behavior (19), and their COVID relevant decision-making. We assessed individuals' collective orientation (norms) using a measure of individual-collective primacy (20), which entailed 7-item to which people responded on a 1 (highly disagree) to 7 (highly agree) response scale (α = 0.59). One additional item used in the original scale was omitted as it reduced the reliability of the full scale (i.e., “*In most cases, to cooperate with someone whose ability is lower than yours is not as desirable as doing the thing on your own”*). Participants attitudes were assessed using two items (*r* = 0.25, *p* < 0.001) regarding individual vs. collective orientation (i.e., “*It is best to quarantine the entire population to save those who are at risk (such as the elderly)”*; “*Concern for the environment is more important than concern for the needs of the individual”*). Planned behavior of adherence to COVID-19-related guidelines was assessed using five statements (α = 0.89) such as: “I intend to strictly make sure to wear a mask.” Finally, participants' decision making was assessed by choosing one of four masks to buy. Participants read that all four masks were identical in terms of the wearer's safety, but they differed in cost (about 0.75, about 1.5, about 3, about 6 USD per unit) and the level of protection to other people they will come in contact with (low, mediocre, good, and excellent). All relevant scales are reported here, and the full scales are available in the Supplementary Materials.

### Results and Discussion

**Figure 2:**
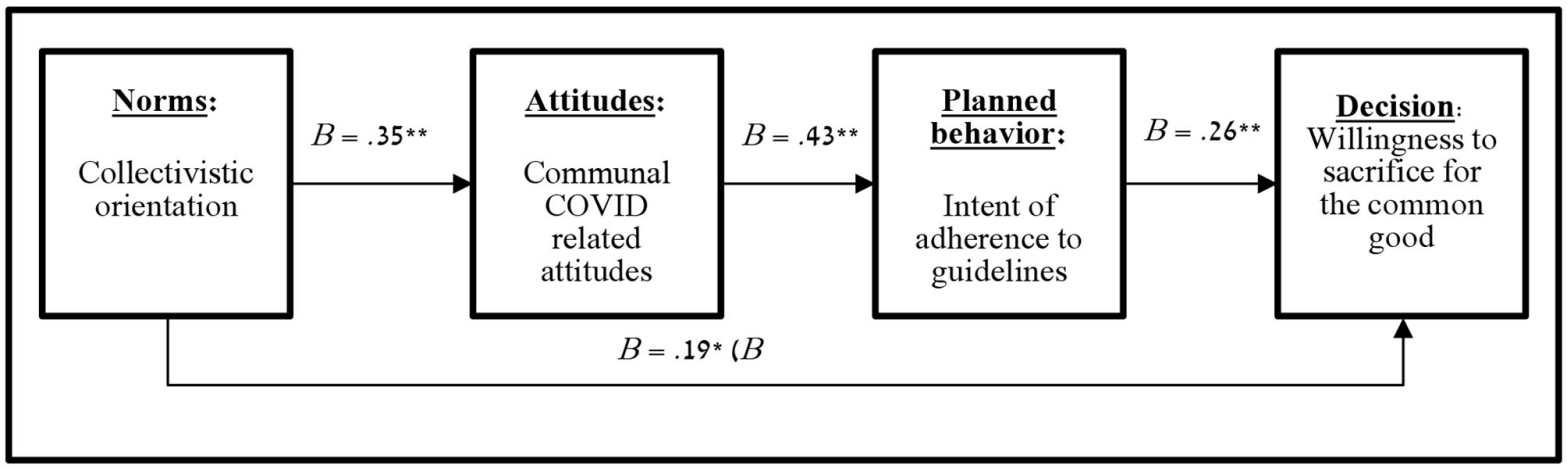
Serial mediation model of collectivism norms, collective attitudes, COVID-19 planned behavior, and relevant decision-making (Study 2). *p < 0.05, **p < 0.01.

We conducted a serial mediation which employed Hayes' (2018 version 3.3;21) PROCESS bootstrapping command (model 6: 5,000 iterations) (21). The effect of each level in the serial mediation was indeed significant (see Figure 2), and the total effect of collectivistic orientation on willingness to make a financial sacrifice for the common good was significant (B = 0.19, SE = 0.08, *p* = 0.021, CI 95% [0.03, 0.36]). The model revealed a full mediation as the direct effect turned insignificant when the indirect path was presented (B = 0.10, SE = 0.08, *p* = 0.240, CI 95% [−0.06, 0.26]). We also found an indirect effect of collectivistic orientation on willingness to sacrifice for the common good via both communal COVID related attitudes and intent of adherence to guidelines (B = 04, SE = 0.02, CI 95% [0.01, 0.08]; total indirect effect: B = 0.10, SE = 0.03, CI 95% [0.04, 0.17]).

The results of Study 2 indicate that collectivistic orientation is associated with willingness to sacrifice for the common good by promoting the protection of one's environment from being infected via communal COVID related attitudes and intent of adherence to COVID health guidelines. The results correspond with the findings of Study 1. These results point to a possible mechanism that may explain the link between collectivistic culture with the number of COVID related cases and, therefore, also deaths found at the state level.

## Study 3

Study 3 was designed to further establish the relations between collectivistic orientation and adherence to health guidelines using different measures among a different sample population. While Study 2 was held in Israel with a sample compiled of mainly Jews, Study 3 was held among American participants.

### Methods

#### Sample

Our sample consisted of 121 American participants (73.6% women; Mage = 27.00, SD = 7.46). Participants were contacted via a large online survey platform (“Prolific”) and were asked to participate in the survey for monetary compensation. Of the participants, the majority (76.9%) were White, and the rest were Black or African American (8.3%), Asian (9.1%), Hispanic or Latino (2.5%), Arab (0.8%), and Multiracial (2.5%). Religion wise, over a quarter of participants were Christians (27.3%), and the rest were Jewish (0.8%), Muslim (6.6%), Hindu (1.7%), Buddhist (1.7%). The rest of the participants defined themselves as Agnostic, Atheist, or Other (62%).

#### Procedure

Here, collectivistic orientation was assessed using three relevant indices: Social-Value orientation (SVO), Perspective-taking (PT), and Empathic concern (EC). SVO was operationalized as the sum of prosocial choices made in a nine-item SVO scale (22). The SVO scale included scenarios in which one has to choose a resource allocation between oneself and another player: equal distribution (prosocial), maximizing one's profit, or maximizing the gap (α = 0.83). A particular example might be (self, other): 480-480, 540-480, and 480-80. Perspective-taking (PT) and Empathic concern (EC) were assessed on a 7-point scale using the sum of participant's responses to 7 items each (23). Among the perspective-taking items was: “*When I'm upset at someone, I usually try to 'put myself in his shoes' for a while”* (α = 0.82). Among the empathic concern items was “*When I see people being taken advantage of, I feel kind of protective toward them”* (α = 0.80). Finally, guideline adherence was assessed by using a single statement: “*Since the COVID-19 eruption, I have been very strict about following the instructions (staying at home, reducing contact with people as much as possible).”*

### Results and Discussion

We tested the correlations among the various manifestations of collectivistic orientation (SVO, PT and EC) to guidelines adherence and found significant correlations. SVO (*r* = 0.21, *p* = 0.022), PT (*r* = 0.34, *p* < 0.001), and EC (*r* = 0.31, *p* = 0.001), were all positively correlated with guidelines adherence.

In Study 3, we replicated the association between collectivistic orientation and guideline adherence by using other measures evaluating collectivistic orientation (not used in Study 2). We had also used an American sample vs. the Israeli sample used in Study 2. The sample was a random (not representative) sample of Prolific participants, which entailed a relatively young and mostly female participants. The correlations found in Study 3 are small to moderate; however, they indicate an effect that may not be of large proportions but is of great importance as it affects the number of human lives lost in the pandemic. Despite the non-representative sample and the moderate size of the correlations, since the results suit the results found in Studies 1 and 2, while using different measures and sample study 3 adds credence to the general argument of this paper.

## Discussion

Some countries suffer a devastatingly high COVID-19 related death toll while others are less affected (4). One cultural aspect that may explain the disparity in fatalities among different countries is the public cooperation and willingness to sacrifice to support the common good and adhere to health guidelines (24). In three studies, we found a tie between individualism (vs. collectivism) to epidemic prevention measures at the personal level (Studies 2 and 3) and a relation between countries' individualism (vs. collectivism) and the mortality rate they suffered at the societal level (Study 1). It is important to note that despite the overall trend we found in Study 1, there may be country-specific differences in the underlying mechanisms that should be further explored moving forward.

The research described in this paper has two main implications. First, for scientists and practitioners examining social aspects of the pandemic, our results suggest that despite the virus outbreak being a global phenomenon, different countries and cultures may react differently to it. Thus, research insight and policy formulation should be treated in a case-by-case manner based on culture, and overarching global generalization should be avoided.

The second implication is that leaders should try to foster a more collectivistic mindset among their constituents regarding promoting safe conduct during the current pandemic or future ones. For example, when trying to promote safe behavior during the pandemic, New York's Governor Andrew Cuomo was quoted saying: “Yeah it's your life do whatever you want, but you are now responsible for my life…. We started saying, It's not about me it's about we.” (25). Alternatively, in cases where the individualistic tendencies are deeply rooted, it might be better to stress the individual benefits of safe conduct and vaccination instead of making the case of collectivistic social responsibility (26). Notably, both approaches should be further investigated to avoid a “boomerang effect,” where counterproductive results might occur, when psychological interventions imply negative social connotations and threaten one's positive self-image (27).

Furthermore, as COVID-19 vaccines have been recently approved, governments and health authorities are now facing a new challenge, namely: people who are reluctant to take the new vaccines out of fear or as part of the anti-vaccine movement (28). Indeed, it seems that even the devastating impact of the COVID-19 pandemic has not convinced those who oppose vaccination (29). Research has pointed to differences in acceptance rates of COVID-19 vaccines across different countries (30). Thus, messages that speak to ones' responsibility toward the community might be more effective within collectivistic communities. Within individualistic societies, on the other hand, self-protection messages should be considered.

To conclude, we argue that cultural variance across countries is just as crucial in understanding adherence to epidemic prevention measures and, therefore, how susceptible a society is to the COVID-19 outbreak. These are initial indications of one mechanism that may explain the disparity of the death toll brought on different cultures by COVID-19.

## Data Availability Statement

All data is available in the main text or as part of the datasets. The datasets generated for this study can be found in the OSF website https://osf.io/8jw2g/?view_only=f7933c6632c84fe383c822b354918fc9.

## Ethics Statement

The studies involving human participants were reviewed and approved by The Institutional Review Board, IDC Herzliya Adelson School of Entrepreneurship. The patients/participants provided their written informed consent to participate in this study.

## Author Contributions

YM: study design, data interpretation, writing, figures, and literature search. AL: study design, data interpretation, and writing. TG: data collection, data analysis, writing results, and figures. DC: data collection, data analysis, and writing results. SS: data collection and data analysis. All authors contributed to the article and approved the submitted version.

## Conflict of Interest

The authors declare that the research was conducted in the absence of any commercial or financial relationships that could be construed as a potential conflict of interest.
